# Are there any differences between age groups regarding colorectal surgery in elderly patients?

**DOI:** 10.1186/1471-2482-14-44

**Published:** 2014-07-15

**Authors:** Huseyin Yuce Bircan, Bora Koc, Umit Ozcelik, Gokhan Adas, Servet Karahan, Alp Demirag

**Affiliations:** 1 Department of Surgery, Baskent University, Faculty of Medicine, Istanbul Research Hospital, Oymacı sokak No:7 Altunizade, Istanbul, Turkey; 2 Department of Surgery, Okmeydani Training and Research Hospital, Istanbul, Turkey

**Keywords:** Elderly Patients, Colon Surgery, Aging

## Abstract

**Background:**

Surgical procedures with curative or palliative intentions in subjects aged over 70 represent a colorectal surgical challenge due to the issue they raise: Benefits versus increased morbidity. In this study, we proposed to compare the impact of surgery with the surgical intervention short-term results and analyze the factors that may influence these results in elderly age groups.

**Methods:**

We retrospectively analyzed a database containing information about patients who underwent colorectal surgery from January 2008 to December 2013 at the Baskent University Istanbul Research Hospital and the Okmeydani Training and Research Hospital.

**Results:**

A total of 265 patients were enrolled and analyzed in this retrospective study. Of these patients operated during the study period, 110 were between 60 and 69 years of age (group 1), 99 were between 70 and 79 years of age and 56 were older than 80 years of age. In total, there were 138 (52%) men and 127 (48%) women that underwent colorectal surgery. Intraoperative complications did not differ between group 1 and group 2, group 2 and group 3; however, some differences were observed between group 1 and group 3 (p = 0.001). Systemic complications were more frequent in group 3 than in groups 1 (p = 0.039) and 2 (p = 0.002). Furthermore, there were no significant systemic complication differences between groups 1 and 2. The mean length of postoperative hospital stay was 9.91 ± 2.65 days in the first group, 9.38 ± 2.44 days in the second group and 11.8 ± 4.35 days in the third group.

**Conclusion:**

Colon surgery for both malignant and non-malignant diseases can be performed safely in different elderly age groups; thus, age should not be considered as an obstacle in elderly patients undergoing colorectal resection.

## Background

Life expectancy is increasing along with the number of elderly patients that undergo surgical procedures. Surgical procedures with curative or palliative intentions in subjects aged over 70 represent a colorectal surgical challenge due to the issue they raise: Benefits versus increased morbidity [1]. The associated comorbidities in elderly patients, such as pulmonary and cardiovascular issues in particular, are mainly responsible for the increased morbidity and mortality [2]. Because surgical techniques and multimodality treatments have improved over the years, postoperative complications after colorectal surgery have decreased gradually.

The indication for surgery in elderly subjects is not dependent upon the patients’ age but by the identification and correction of the known preoperative risk factors that may determine the higher complication or mortality rates [3]. The selected procedures may lead to prolonged hospital stay, increased medical costs and resource overuse due to the aggressiveness of the approach and the high risk of developing postoperative nutritional disorders [4].

The average life expectancy in many developed countries is reported to be more than 80 years; therefore, the issue regarding the treatment of elderly patients with colorectal diseases may be increased [5]. Age classification varies between countries and over time, which reflects social class differences or functional abilities related to the workforce in many instances. The survival rate and quality of life after colorectal surgical treatment are affected by postoperative complications. In this study, we proposed to compare the impact of surgery with the surgical intervention short-term results and analyze the factors that may influence these results in elderly age groups.

## Methods

We retrospectively analyzed a database containing information about patients who underwent colorectal surgery from January 2008 to December 2013 at the Baskent University Istanbul Research Hospital and the Okmeydani Training and Research Hospital. The study was reviewed and approved by the Institutional Review Board of the Okmeydani Training and Research Hospital. Patients were divided into three groups according to age: group 1 patients were aged between 60–69; group 2 patients were aged between 70–79 and; group 3 patients were aged older than 80. Experienced surgeons performed all of the operations and none of the patients received any additional treatments, including chemo-radiotherapy. The patients in which emergency surgery was required for accompanying complications, such as perforation or multi-organ failure, and cases in which a curative resection could not be performed were excluded from the study.

Expert anesthesiologists conducted a preoperative risk evaluation by using the American Society of Anesthesiology (ASA) classification. For antibiotic prophylaxis, all patients received a dose of cephalosporin during their anesthesia induction. A second dose of the same antibiotic was administered if the surgery lasted more than four hours. Deep vein thrombosis prophylaxis was performed with low-molecular-weight heparin (4000 IU/day) in all patients 8 hours before the elective surgery or 8 hours after the emergency surgery.

The patients’ preoperative characteristics were obtained, including age, gender, diagnosis and comorbidities. The surgical technique type, length of postoperative hospital stay, pathological differentiation and clinical malignancy stage, as well as morbidity and mortality were analyzed to compare the surgery risks and benefits in the three groups. The surgical procedures were conducted with respect to the surgical findings, and the tumor location was recorded.

The postoperative bowel function was also evaluated with respect to the first flatus and bowel movements. Clear liquid was started after first flatus, provided that the patient did not have severe nausea, vomiting or abdominal distention. The postoperative morbidity was defined as complications that required additional treatment or prolonged the hospital stay. The operative mortality was defined as death within 30 days of surgery.

### Statistical analysis

All calculations were performed with the SPSS software package, version 16.0. The categorical data are presented as frequencies and percentages compared by the Chi-square test. The parametric and nonparametric continuous data are presented as the means and standard deviations and were evaluated by the Mann–Whitney U test. A *p*-value of 0.05 was considered as a statistically significant value.

## Results

A total of 265 patients were enrolled and analyzed in this retrospective study. Of these patients operated during the study period, 110 were between 60 and 69 years of age (group 1), 99 were between 70 and 79 years of age and 56 were older than 80 years of age. In total, there were 138 (52%) men and 127 (48%) women that underwent colorectal surgery. The patients’ demographic parameters, ASA scores, comorbidities, presentation onset symptoms and diagnostic procedures are listed in Table 1. The median ages were 63.58 ± 2.52 in group 1 (range, 60 to 69 years), 74.39 ± 2.59 in group 2 (range, 70 to 79 years) and 85.63 ± 3.55 in group 3 (range, 80 to 97 years). As expected, group 2 and especially group 3 presented with significantly higher average ASA scores and comorbid conditions. In particular, a higher proportion of diabetes mellitus (DM), respiratory disease and cerebrovascular disease were observed in group 3. There were not any cardiovascular disease differences between the groups. The most common causes of admission to the hospital were blood in the stool for group 1, bowel obstruction for group 2 and anemia for group 3.

**Table 1:** The patients’ demographic parameters, ASA scores, comorbidities, presentation onset symptoms and diagnostic procedures are listed

	**Group 1**	**Group 2**	**Group 3**
**Sex**			
**Male/Female**	52/58	43/56	32/24
**Age**			
**ASA**	32	22	7
**I**	46	39	12
**II**	21	26	21
**III**	11	12	16
**IV**			
**Comorbidity**	87 (79%)	73 (74%)	43 (77%)
**Cardiovascular**	34 (30%)	24 (24%)	21(37.5%)
**DM**	21 (19%)	12 (12%)	16(28.5%)
**Respiratory Disease**	8 (7.2%)	5 (5%)	9 (16%)
**Cerebrovascular Disease**	12 (11%)	17 (17%)	14 (25%)
**Others**			
**Presentation**	18 (16%)	21 (21%)	12 (21%)
**Asymptomatic**	92 (84%)	78 (79%)	44 (79%)
**Symptomatic**	14 (12%)	23 (23%)	13 (23%)
**Obstruction**	22 (19%)	17 (17%)	16 (28%)
**Anemia**	30 (30%)	21 (21%)	11 (19%)
**Blood in Stool** **Weight Loss**	26 (23%)	17 (17%)	4 (7%)
**Diagnostic**	23 (21%)	32 (32%)	16 (28%)
**Procedure**	78 (70%)	60 (61%)	32 (57%)
**Computerized Tomography**	9 (8%)	7 (7%)	8 (15%)
**Colonoscopy Exploration**			

The surgical procedures used and pathological characteristics of the resected specimens are also shown in Table 2. After resecting the lesion, a double or single-stapler technique, hand-sutured anastomoses and functional end-to-end anastomoses were performed for reconstruction in both groups. Moreover, colostomies were performed after the Mile’s approach. There were no statistically significant differences between the groups regarding the reconstruction methods. Additionally, most of the specimens were diagnosed as malignant lesions after pathologic evaluations. The group 1 tumor stages were as follows: stage I in 1 patient, stage II in 28 patients, stage III in 53 patients and stage IV in 13 patients. In group 2, the tumor stages were as follows: stage II in 27 patients, stage III in 47 patients and stage IV in 6 patients. In group 3, the tumor stages were as follows: stage II in 14 patients, stage III in 25 patients and stage IV in 1 patient.

**Table 2:** The surgical procedures used and pathological characteristics of the resected specimens are shown

	**Group 1**	**Group 2**	**Group 3**
Operations	**30**	**25**	**16**
Right Hemicolectomy	**3**	**2**	**2**
Transvers Colectomy	**35**	**19**	**15**
Left Colectomy	**7**	**14**	**9**
Expanded Right Hemicolectomy	**22**	**20**	**6**
Anterior Resection	**7**	**8**	**3**
Low Anterior Resection	**7**	**4**	**1**
Abdominoperineal Resection	**4**	**7**	**4**
Total/Subtotal Colectomy			
Postoperative Diagnosis	**110**	**99**	**56**
Cancer	**95**	**79**	**40**
Inflammation	**5**	**10**	**6**
Ischemia	**10**	**10**	**10**
Stage	**1**	**0**	**0**
I	**18**	**17**	**6**
II	**9**	**10**	**6**
2a	**1**	**0**	**2**
2b	**4**	**4**	**1**
2c	**25**	**18**	**13**
III	**24**	**25**	**11**
3a	**11**	**5**	**1**
3b	**2**	**1**	**0**
3c			
IV			
4a			
4b			

Intraoperative complications did not differ between group 1 and group 2, group 2 and group 3; however, some differences were observed between group 1 and group 3 (p = 0.001) (Table 3). Although there were no significant postoperative surgical complication differences between the three groups, there were significantly different systemic complications (non-surgical) between the groups. Systemic complications were more frequent in group 3 than in groups 1 (p = 0.039) and 2 (p = 0.002). Furthermore, there were no significant systemic complication differences between groups 1 and 2.

**Table 3:** The intraoperative and postoperative complications are detailed in table

	**Group 1**	**Group 2**	**Group 3**
Intraoperative Complications	**2**	**1**	**2**
Operative hemorrhage ≥500 cc	**1**	**0**	**1**
Organ Injury	**1**	**1**	**1**
Postoperative Complications (surgical)	**11**	**13**	**12**
Anastomotic Leakage	**2**	**2**	**2**
Postoperative Hemorrhage	**1**	**1**	**1**
Wound Infection	**5**	**5**	**4**
Incisional Hernia	**2**	**3**	**3**
Prolonged Ileus	**1**	**2**	**2**
Postoperative Complications	**6**	**12**	**17**
(non-surg)	**1**	**2**	**6**
Cardiovascular	**0**	**1**	**1**
Renal	**1**	**3**	**4**
Pneumonia	**0**	**1**	**2**
Neurological	**2**	**4**	**1**
Urinary Tract Problems	**2**	**1**	**2**
Pulmonary embolism			
Hospitalization Period (day)	**9.91 ± 2.65**	**9.38 ± 2.44**	**11.8 ± 4.35**
Mortality	**5**	**3**	**4**
AMI	**0**	**1**	**2**
Anastomosis Leakage	**2**	**1**	**1**
Pneumonia	**1**	**1**	**1**
Pulmonary embolism	**2**	**0**	**0**

The mean length of postoperative hospital stay was 9.91 ± 2.65 days in the first group, 9.38 ± 2.44 days in the second group and 11.8 ± 4.35 days in the third group. Although the comorbidity rate was similar in the groups, the length of postoperative hospital stay was significantly longer in group 3 than in groups 1 (p = 0.009) and 2 (p = 0.010). Additionally, there was no significant difference in the operation related morbidity between three groups.

## Discussion

Populations around the world are rapidly aging. According to the WHO, the proportion of people over 60 years of age is increasing faster than any other population in nearly every country and is expected to surpass 2 billion people worldwide by the year 2050 [6]. The term ‘elderly’ is a very subjective term. The term is based on the interpretation of the culture in which the person lives. Conventionally, “elderly” has been defined as a chronological age of 65 years old or older. Further, those who are 65 through 74 years old are referred to as “early elderly” and those over 75 years old are known as “late elderly.” The evidence upon which this definition is based is unknown, however. In this current study, we compared the short-term colorectal surgical treatment outcomes in three consecutive elderly age groups.

Even in developing countries, most older people die of non-communicable diseases such as heart disease, cancer and diabetes, rather than from infectious and parasitic diseases. Additionally, older people often have several coexisting health problems, such as diabetes and heart disease. Elderly patients represent a high percentage of patients diagnosed and treated for colon surgery due to their progressive increase in life expectancy with the consequent aging population [3]. Health status is an important factor that has a significant impact on the quality of life and mortality on the elderly population after surgery. Although the preoperative statuses, as evaluated by the ASA scoring system, were worse in group 3 than in groups 1 and 2, almost all of the patients could be treated with surgery in this study. A study from the USA reported that arthritis was the most prevalent (48.9%) chronic condition among elderly patients ≥ 65 years, followed by hypertension (40.3%) [7]. In the present study, cardiovascular disease was the most common disease (77-79%) among the observed elderly patients followed by DM (30–37.5%). However, there were no cardiovascular disease differences between the groups; however, more DM, respiratory disease and cerebrovascular disease diagnoses were made for group 3 compared with group 1 and 2.

The most frequent symptoms that patients present to hospitals are usually different for various age groups. Mahdi et al. reported that the most frequent symptoms were per-anal bleeding (35%), weakness (25%), constipation (18%), a recent change in bowel habits (14.2%) and paradoxical diarrhea (9%) for patients with colorectal malignancies [8]. Additionally, only abdominal pain was more frequent in patients under 75 years of age. Parallel to this literature, the cause of hospital admission was different in all three groups. The most common cause of admission to the hospital was blood in the stool in the younger than 70-year-old patient, bowel obstruction in the patients between 70–80 and anemia in the patients that were greater than 80 years of age. This difference might be due to the heterogeneity of our study group (which contained both malignant and non-malignant colon surgeries). Moreover, colorectal cancers in older patients are more likely revealed by a complication, this fact is related to a delay in the diagnosis in older patients and this is due to negligence of symptoms in these patients [9].

Knowledge regarding the colorectal surgical management in older patient’s is rapidly expanding. Several studies have shown that age alone does not influence outcomes after surgery for colorectal diseases, but it is rather the comorbidity and compromised physical capacity to recover from adverse events that may occur in connection with major surgery [10]. Schwander et al. divided patients into the following age-related groups: patients 50 years of age or younger, patients ranging from 51 to 70 years of age, and patients older than 70 years of age [11]. They observed no statistically significant major and minor complication differences among the three groups. Nevertheless, controversy continues because other authors have reported a somewhat higher complication rate in elderly patients [12-15]. Additionally, they observed that postoperative hospitalizations were significantly prolonged in patients older than 70 years. In our study, there was only a significant intraoperative complication difference between groups 1 and 3. We think that this difference was dependent upon surgical technique difficulties and not age-related complications. Despite the fact that no statistically significant difference was found between the three age groups, patients aged more than 80 years presented greater postoperative complications. It is also of note that postoperative complications related to surgery developed in only 12 patients from the group 3 elderly patient cohort (21.4%). The non-surgical complication incidence increased significantly with age. In the elderly patient group (≥80 years) we have objectified a higher percentage of non-surgical post-operative complications, mostly due to a higher number of cardiovascular disease occurrences, as well as general postoperative complications caused by pneumonia. As a result, the systemic complications were more frequent in group 3 than in groups 1 and 2. Furthermore, there were no systemic complication significant differences between group 1 and 2. Hence, the term “elderly”, which is defined as older than 65 years of age, may be mentioned as a threshold of older than 80 years of age in colon surgery instances.

As previously reported, the hospital length of stay for elderly colorectal surgery patients was the same as that in younger patients [2,16,17]. Senagore et al. reported there was no difference between patients 70 years old or older who underwent colorectal surgery compared with patients younger than 60 years of age [18]. In the current study, the group 3 patients (those older than 80 years) had longer hospital stay durations than the other groups. This was the result of excess of comorbidities in the patients older than 80 years of age.

The present study was subject to several limitations. First, it was not a randomized controlled study; therefore, a prospective randomized controlled study is needed to demonstrate that colon surgery in elderly patients is truly a feasible procedure for different older age groups. Additionally, the indication of colon surgery was not distinguished between malignant and non-malignant diseases in this study.

## Conclusion

In conclusion, colon surgery for both malignant and non-malignant diseases can be performed safely in different elderly age groups; thus, age should not be considered as an obstacle in elderly patients undergoing colorectal resection.

## Abbreviations

ASA: American Society of Anesthesiology; DM: Diabetes mellitus.

## Competing interests

The authors declare that they have no competing interests.

## Authors’ contributions

HYB, UO, GA, SK, AD and BK analyzed the data and wrote the initial draft. HYB and BK edited manuscript with literature review. All authors read and approved the final manuscript.

## Pre-publication history

The pre-publication history for this paper can be accessed here:

http://www.biomedcentral.com/1471-2482/14/44/prepub
